# Reparo primário artroscópico do ligamento cruzado anterior com fixação femoral em EndoButton ajustável e reforço dinâmico com sutura de alta resistência

**DOI:** 10.1055/s-0045-1814431

**Published:** 2026-03-24

**Authors:** Antonio Carlos Moscon, Fabrício Luz Cardoso, Daniel Rocha de Almeida Braga, Claudio Gattás

**Affiliations:** 1 Grupo de Cirurgia do Joelho, Clínica Ortocity – Ortopedia, Fraturas e Reabilitação, São Paulo, SP, Brasil

**Keywords:** artroscopia, ligamento cruzado anterior, técnicas de sutura, anterior cruciate ligament, arthroscopy, suture techniques

## Abstract

O reparo primário do ligamento cruzado anterior (LCA) tem ressurgido como alternativa em casos selecionados, impulsionado pelo avanço das técnicas artroscópicas e pelo desenvolvimento de dispositivos modernos de fixação. Este trabalho descreve a técnica de reparo artroscópico do LCA com fixação femoral em EndoButton (Smith & Nephew) ajustável associada ao reforço dinâmico com sutura de alta resistência. A indicação mais favorável ocorre em rupturas proximais agudas (tipos I e II de Sherman et al.), nas quais há bom remanescente ligamentar e potencial de cicatrização. O procedimento visa preservar a anatomia e a propriocepção originais, reduzir a morbidade da retirada de enxertos e manter opções futuras de reconstrução. A técnica inclui sutura do coto ligamentar, fixação femoral ajustável e reforço interno, o que proporciona estabilidade biomecânica adicional. O protocolo pós-operatório proposto, baseado no fundamento lógico da estabilidade, privilegia mobilização precoce, carga progressiva e reabilitação estruturada. Esta abordagem representa uma alternativa promissora para pacientes criteriosamente selecionados, sobretudo atletas jovens e indivíduos em crescimento, desde que respeitadas as indicações e as limitações da técnica, sendo necessária a validação por estudos de longo prazo para a consolidação de sua eficácia clínica.

## Abstract (en)

Primary anterior cruciate ligament (ACL) repair has reemerged as an alternative in selected cases thanks to advances in arthroscopic techniques and the development of modern fixation devices. The present paper describes the arthroscopic ACL repair technique with femoral fixation using an adjustable EndoButton (Smith & Nephew) combined with dynamic reinforcement using a high-strength suture. This technique is primarily indicated for acute proximal ruptures (Sherman et al. types I and II), which present a good ligament remnant and healing potential. The procedure aims to preserve the original anatomy and proprioception, reduce the morbidity associated with graft harvesting, and maintain future reconstruction options. The technique involves suturing the ligament stump, using adjustable femoral fixation, and applying internal bracing to provide additional biomechanical stability. The proposed postoperative protocol, based on the rationale of stability, prioritizes early mobilization, progressive weight-bearing, and structured rehabilitation. This approach represents a promising alternative for carefully-selected patients, especially young athletes and skeletally-immature subjects, provided the indications and limitations of the technique are respected. Long-term studies are needed to consolidate the clinical efficacy of this technique.

## Introdução

O interesse no reparo primário do ligamento cruzado anterior (LCA) nas últimas duas décadas decorre dos avanços nas técnicas artroscópicas e de evidências clínicas que demonstram a capacidade de cicatrização confiável do LCA em casos selecionados, com taxas de retorno ao esporte comparáveis às da reconstrução do LCA (RLCA). 1 2 3

O fundamento lógico do reparo primário do LCA reside nos potenciais benefícios de preservação da propriocepção, na manutenção da anatomia original do ligamento e na menor agressão óssea decorrente da confecção de túneis menores ou de menos túneis. Essa estratégia também visa eliminar complicações associadas à retirada de enxertos autólogos, como dor anterior no joelho, fraqueza dos músculos isquiotibiais ou do quadríceps, câimbras e risco de ruptura dos tendões remanescentes. 2 4

O aprimoramento tecnológico, com o surgimento de fios de alta resistência e sistemas de suspensão ajustáveis, possibilitou a associação do reparo anatômico a técnicas de reforço interno ( *augmentation* ). Essa combinação busca favorecer a cicatrização biológica, amplia a segurança biomecânica e contribui para uma expectativa de recuperação funcional mais precoce, com ganho de amplitude de movimento e sensação subjetiva de joelho mais natural. 5 6

A classificação de Sherman et al., 7 que identifica lesões proximais do LCA como mais propensas à cicatrização, foi um marco importante na pesquisa sobre a RLCA.

Estudos recentes 1 6 relatam taxas de rerruptura entre 7 e 20% em lesões proximais (tipos 1 e 2 de Sherman et al. 7 ), atualmente as mais investigadas. Esses dados sustentam o reparo primário como alternativa viável em cenários específicos, embora ainda haja necessidade de estudos de seguimento prolongado para a validação definitiva da técnica.

Este artigo tem como objetivo descrever detalhadamente a técnica artroscópica de reparo primário do LCA com fixação femoral em EndoButton (Smith & Nephew) ajustável e reforço dinâmico com sutura de alta resistência. Os detalhes do procedimento foram documentados e refinados em um modelo cadavérico *fresh frozen* para facilitar a demonstração do passo a passo. O trabalho apresenta os cuidados pós-operatórios propostos e discute o fundamento lógico biomecânico da técnica, e ressalta suas vantagens teóricas em relação às técnicas convencionais com implante externo.

## Seleção dos pacientes

O sucesso do reparo primário do LCA depende da seleção criteriosa dos pacientes. A avaliação inicial deve incluir histórico clínico detalhado, exame físico completo e análise de exames de imagem.

Pacientes com ruptura do LCA frequentemente relatam um estalo agudo no momento da lesão, seguido de hemartrose e instabilidade subjetiva do joelho, resultante da translação tibial anterior não resistida pelo ligamento rompido. 8

Sempre que possível, deve-se avaliar, no exame físico, a frouxidão ligamentar por meio dos testes de Lachman e *pivot shift* , que apresentam alta especificidade para a detecção da instabilidade tibial anterior e rotatória. Radiografias-padrão podem auxiliar na identificação de fraturas associadas, como a fratura de Segond, ao passo que a ressonância magnética (RM) é fundamental para confirmar o diagnóstico, avaliar o remanescente ligamentar e detectar lesões concomitantes.

As indicações ideais incluem rupturas proximais agudas, particularmente avulsões femorais de tipo I de acordo com a classificação de Sherman et al., 7 associadas a bom remanescente ligamentar e intervalo curto entre lesão e reparo, fatores que favorecem maior potencial de cicatrização e resultados clínicos satisfatórios. 6 8 9 Pacientes pediátricos ou em fase de crescimento também se beneficiam da preservação biológica do LCA, desde que a integridade do coto ligamentar seja mantida. 9

Em contrapartida, lesões crônicas, rupturas no terço médio do ligamento ( *mid-substance* ) ou distais, bem como tecido degenerado e recidivas em ligamento previamente reparado, representam contraindicações, pois comprometem a qualidade biomecânica do reparo e elevam os riscos de falha. 9

Mesmo nos casos de suspeita de ruptura proximal evidenciada por imagem, a decisão definitiva entre reparo primário e reconstrução deve ser realizada intraoperativamente **,** com avaliação direta do tipo de ruptura e da qualidade do tecido, discutindo previamente as alternativas de enxerto caso a reconstrução seja necessária.

Entre populações especiais, como atletas jovens de esportes que envolvem movimentos de pivô e adultos com alta demanda física, o reparo primário pode ser uma alternativa viável em situações selecionadas. Contudo, a literatura 10 recomenda avaliação criteriosa, indicação prudente e reabilitação estruturada, visto o risco aumentado de reruptura nesses grupos.

## Descrição da técnica

### Posicionamento

O paciente é posicionado em decúbito dorsal, sob anestesia geral ou raquidiana, com torniquete pneumático no terço proximal da coxa. O membro a ser operado é apoiado em suporte lateral, o que permite ampla flexoextensão. Profilaxia antibiótica é administrada conforme protocolo institucional.

### Acessos e inspeção artroscópica

São confeccionados os portais anterolateral (óptica) e anteromedial (trabalho), com portal anteromedial acessório para a confecção do túnel femoral. Realiza-se inventário articular, com tratamento de lesões meniscais ou condrais associadas.

### Identificação do LCA e preparo do coto

**Fig. 1:**
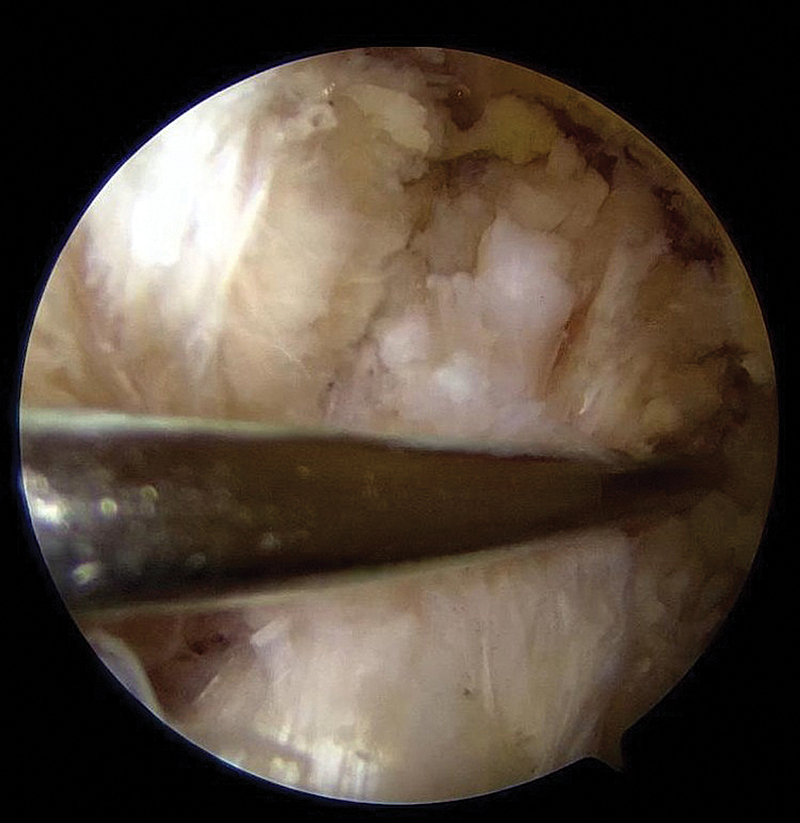
Identificação da lesão proximal do ligamento cruzado anterior (LCA).

A ruptura proximal do LCA é identificada, e preserva-se ao máximo o remanescente ligamentar ( Fig. 1 ). O desbridamento é mínimo, e utiliza *shaver* ou radiofrequência somente para a remoção de fibras instáveis, o que preserva a vascularização. O *footprint* femoral é discretamente cruentado com *shaver* ou cureta para favorecer a cicatrização.

### Sutura do remanescente

**Fig. 2:**
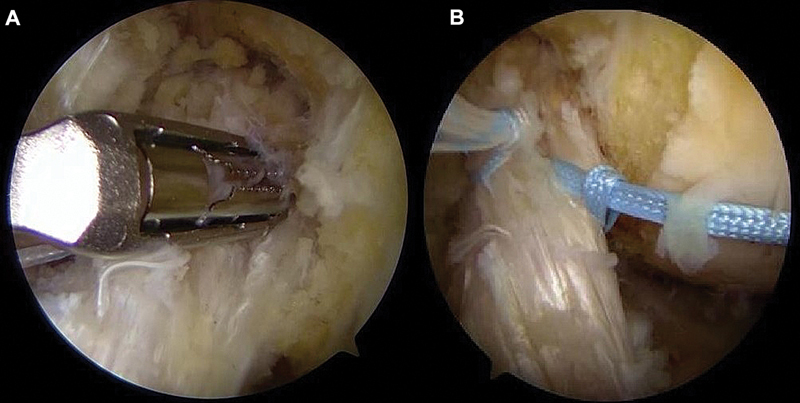
Posicionamento do passa-fio artroscópico ( **A** ). Realização das duas suturas com fios de alta resistência envolvendo os feixes anteromedial e posterolateral ( **B** ).

Com passa-fio artroscópico, realizam-se 2 suturas com fios de alta resistência (de n° 2) feitos de 100% de polietileno em configuração tipo *lasso-loop* ou bloqueada, do distal para o proximal, envolvendo os feixes anteromedial e posterolateral ( Fig. 2 ). Os fios permanecem livres para posterior tensionamento.

### Fixação femoral

**Fig. 3:**
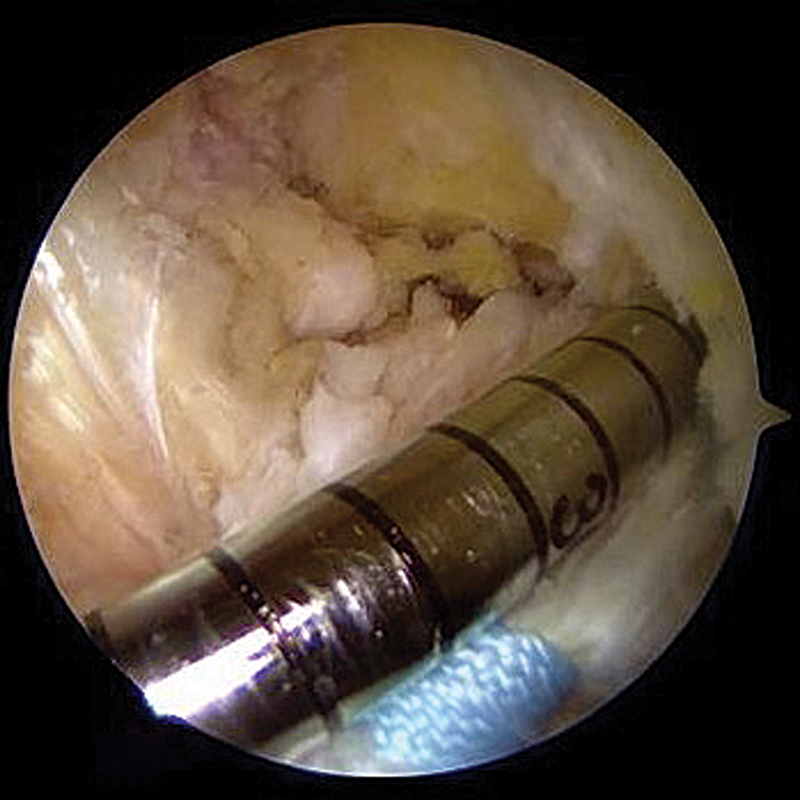
Confecção do túnel femoral no *footprint* anatômico do LCA pelo portal anteromedial acessório com auxílio de broca de 4,5 mm.

**Fig. 4:**
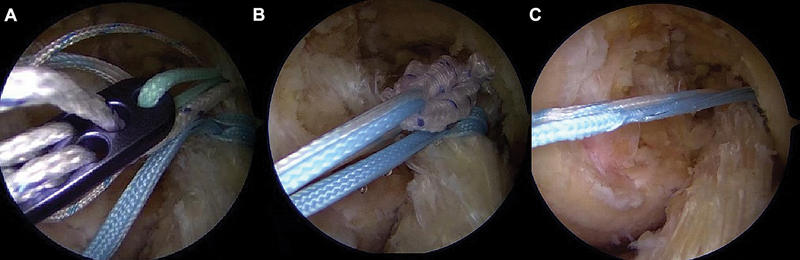
Passagem dos fios de sutura pelo *loop* ajustável do EndoButton ( **A** ). Condução do coto do LCA pelo túnel do fêmur ( **B** ). Fixação em suspensão do LCA reparado na parede medial do côndilo femoral lateral ( **C** ).

Com fio-guia femoral e broca de 4,5 mm, confecciona-se túnel no *footprint* anatômico do LCA pelo portal anteromedial acessório, com hiperflexão do joelho ( Fig. 3 ). Os fios de sutura do coto ligamentar são passados pelo *loop* ajustável do EndoButton e conduzidos pelo túnel, sendo fixados em suspensão cortical. O coto ligamentar é reduzido ao seu *footprint* tracionando-se os fios passados escarranchadamente pelo *loop* ajustável ( Fig. 4 ).

### Reforço ( *internal brace* )

Os mesmos fios utilizados na sutura do coto são empregados como reforço interno, o que oferece suporte adicional durante a cicatrização ligamentar ( Fig. 4 ).

### Fixação tibial e ajuste final

**Vídeo 1** Passo a passo da técnica de sutura primária do LCA.


**Fig. 5:**
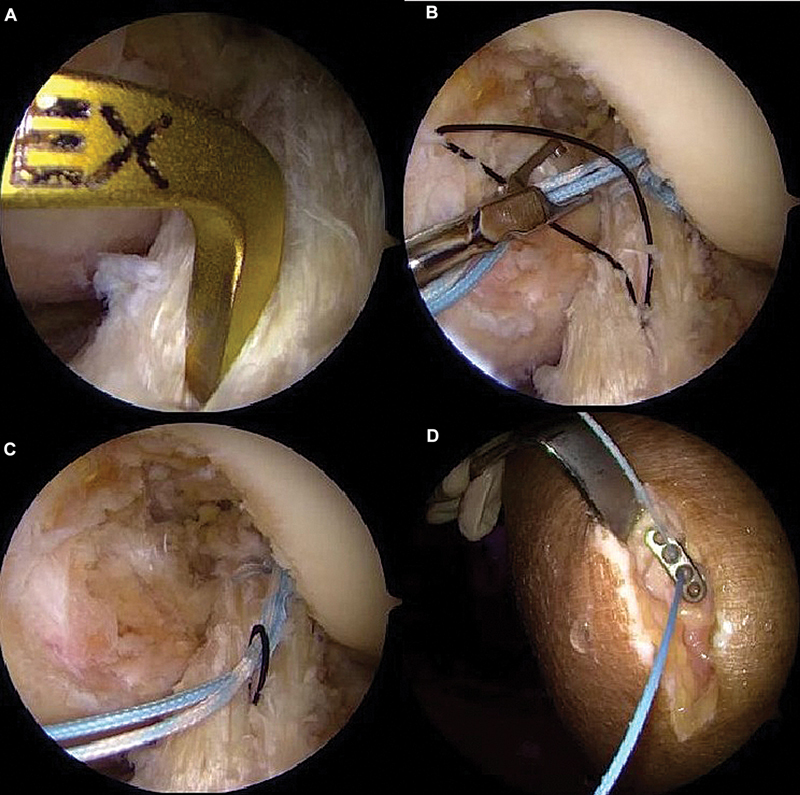
Posicionamento do túnel tibial ( **A** ). Passagem dos fios pelo LCA distal ( **B** ). Direcionamento dos fios do reforço para o interior do túnel da tíbia ( **C** ). Fixação na cortical anterior da tíbia com EndoButton ( **D** ).

Os fios são conduzidos por um túnel tibial de 4,5 mm na posição da inserção da banda anteromedial, e fixados à cortical anterior da tíbia com EndoButton ou âncora *knotless* ( Fig. 5 ). O joelho é posicionado entre 0 e 20° de flexão para o tensionamento adequado do *loop* ajustável.

Por meio de artroscopia de controle são verificadas a isometria, a ausência de *impingement* e a estabilidade após o reparo. Em seguida, são realizadas três a quatro microfraturas na face medial do côndilo femoral lateral, adjacentes ao *footprint* . Esta manobra visa promover um estímulo biológico ( *marrow stimulation* ). 11 A perfuração do osso subcondral permite a liberação de células-tronco mesenquimais e de fatores de crescimento da medula óssea no local do reparo, o que, por analogia a estudos 11 que utilizam concentrados de aspirado de medula óssea ( *bone marrow aspirate concentrates* , BMACs, em inglês) em reconstruções ligamentares, busca acelerar e otimizar a cicatrização e a maturação do tecido ligamentar reparado. Todo o procedimento pode ser visualizado no Vídeo 1 , que demonstra a técnica passo a passo.

### Fechamento

Após irrigação abundante, os portais são fechados com sutura simples de náilon, seguida de curativo compressivo.

### Pós-operatório

O protocolo segue mobilização precoce, carga progressiva conforme tolerância e uso de órtese articulada inicial. O objetivo é estimular a cicatrização biológica sem comprometer a estabilidade articular.

## Conclusão

A técnica descrita combina os princípios do reparo primário anatômico do LCA com o uso de dispositivos modernos de suspensão, e associa reforço interno por meio dos fios de sutura do coto ligamentar passados escarranchadamente pelo *loop* do EndoButton.

Essa configuração proporciona fixação dinâmica, o que promove a aproximação contínua do coto ligamentar ao *footprint* femoral mesmo diante de discreta anteriorização tibial. A escolha do EndoButton ajustável, em detrimento de fixações “fixas”, permite o tensionamento ideal e a regulagem *in situ* do reparo após a fixação cortical. Esta capacidade de ajuste contínuo é fundamental para um reparo com reforço dinâmico, pois confere uma vantagem biomecânica ao sistema, o que previne a perda de tensão que pode ocorrer durante os ciclos de movimento articular e favorece a coaptação constante do coto ao leito biológico.

Trata-se de uma alternativa com grande potencial teórico e biomecânico, que preserva o tecido nativo e mantém a possibilidade de futuras revisões cirúrgicas com menor complexidade técnica.

Embora este manuscrito se classifique como uma “nota técnica”, pois se limita à descrição detalhada do procedimento, a técnica já foi aplicada em 15 pacientes em nossa experiência inicial, mas ainda sem resultados suficientes para análise. Para a validação clínica de seus benefícios, é necessário um seguimento adequado. Neste sentido, planejamos o acompanhamento prospectivo dos pacientes por um período de 6 meses a 2 anos, por meio de reavaliações clínicas e funcionais, utilizando escalas validadas para o joelho (como a do International Knee Documentation Committee) e avaliação por RM em 3, 6 e 12 meses de pós-operatório, visando consolidar os resultados e comparar a eficácia com o padrão-ouro.
